# High‐Performance Poly(1‐naphthylamine)/Mesoporous Carbon Cathode for Lithium‐Ion Batteries with Ultralong Cycle Life of 45000 Cycles at ‐15 °C

**DOI:** 10.1002/advs.202302490

**Published:** 2023-06-09

**Authors:** Junkai Yang, Xiaoru Zhao, Jixing Yang, Yunhua Xu, Yuesheng Li

**Affiliations:** ^1^ School of Materials Science and Engineering Tianjin Key Laboratory of Composite and Functional Materials Tianjin University Tianjin 300072 China

**Keywords:** in situ electropolymerization, lithium‐ion batteries, low‐temperature performance, ordered mesoporous carbon, organic cathode materials

## Abstract

Organic electrode materials for lithium‐ion batteries have attracted significant attention in recent years. Polymer electrode materials, as compared to small‐molecule electrode materials, have the advantage of poor solubility, which is beneficial for achieving high cycling stability. However, the severe entanglement of polymer chains often leads to difficulties in preparing nanostructured polymer electrodes, which is vital for achieving fast reaction kinetics and high utilization of active sites. This study demonstrates that these problems can be solved by the in situ electropolymerization of electrochemically active monomers in nanopores of ordered mesoporous carbon (CMK‐3), combining the advantages of the nano‐dispersion and nano‐confinement effects of CMK‐3 and the insolubility of the polymer materials. The as‐prepared nanostructured poly(1‐naphthylamine)/CMK‐3 cathode exhibits a high active site utilization of 93.7%, ultrafast rate capability of 60 A g^−1^ (≈320 C), and an ultralong cycle life of 10000 cycles at room temperature and 45000 cycles at −15 °C. The study herein provides a facile and effective method that can simultaneously solve both the dissolution problem of small‐molecule electrode materials and the inhomogeneous dispersion issue of polymer electrode materials.

## Graphical abstract

A brand‐new strategy of in situ electropolymerization in nanopores of CMK‐3 is developed to fabricate nanostructured polymer cathode for lithium‐organic batteries, which enables high capacity (close to its theoretical value), ultrafast (60 A g^−1^) and ultralong cycle life at both room temperature (10000 cycles) and low temperature of −15 °C (45000 cycles).

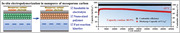

## 1 Introduction

Lithium‐ion batteries (LIBs) have been widely used in various fields of social life, such as electric vehicles and consumer electronic products.^[^ ^1^, ^2^ ^]^ Inorganic materials, including transition metal oxides and metal phosphates, are currently used as cathode materials for commercial LIBs.^[^ ^3^ ^]^ However, the widespread application of LIBs has caused increasing concerns regarding the nonrenewability of transition metal resources and environmental pollution.^[^ ^4^, ^5^, ^6^ ^]^ Organic electrode materials have many advantages such as abundant resources, low cost, environmental friendliness, and structural diversity, and have emerged as promising candidates for next‐generation LIBs.^[^ ^6^, ^7^, ^8^, ^9^, ^10^, ^11^ ^]^ Nevertheless, organic electrode materials often encounter serious dissolution problems in liquid electrolytes, leading to poor cycling stability.^[^ ^12^, ^13^, ^14^ ^]^ Moreover, another shortcoming of most organic electrode materials is their insufficient electronic conductivity, which often leads to inferior rate performance and low active site utilization.^[^ ^10^, ^14^, ^15^ ^]^

**Scheme 1:**
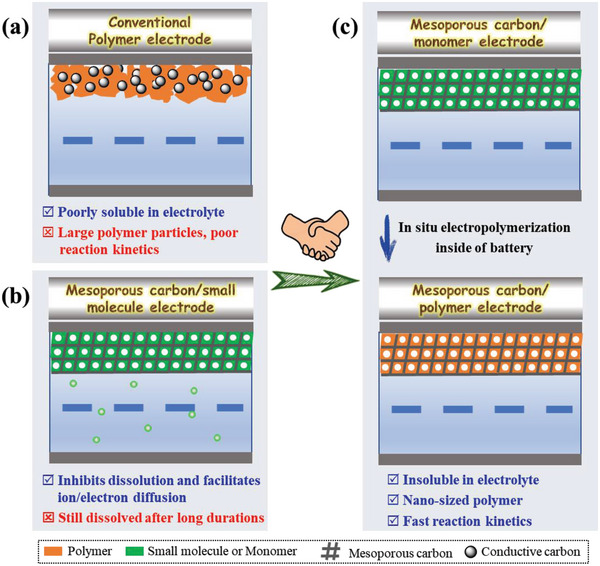
Illustration of previous strategies for addressing the dissolution problems of organic electrode materials by a) capsulizing in CMK‐3 and b) synthesizing polymers. c) Illustration of the proposed strategy for in situ electropolymerization in the nanopores of CMK‐3 inside the battery in this study.

Various approaches have been developed to address the dissolution issue, including the adoption of gel/solid‐state electrolytes^[^ ^16^, ^17^ ^]^ or modified separators,^[^ ^18^, ^19^, ^20^ ^]^ and molecular structure optimization (such as salification,^[^ ^21^, ^22^ ^]^ increasing molecular size,^[^ ^16^, ^23^, ^24^ ^]^ and polymerization^[^ ^25^, ^26^, ^27^, ^28^ ^]^). Among them, synthesis of poorly soluble redox‐active polymers has proven to be an effective way to tackle the dissolution problem.^[^ ^29^, ^30^, ^31^, ^32^, ^33^ ^]^ However, the polymer route still faces considerable challenges, such as complicated synthesis and, importantly, the serious agglomeration of polymer chains.^[^ ^31^, ^33^, ^34^ ^]^ As depicted in **Scheme** **1**a, the severe entanglement of polymer chains usually make it difficult to realize homogeneous dispersion with conductive agents, resulting in more sluggish electron/ion diffusion kinetics and lower active site utilization than that of small molecules.^[^ ^35^ ^]^ Recently, a convenient and efficient in situ electropolymerization method was developed to mitigate these problems.^[^ ^36^, ^37^, ^38^, ^39^ ^]^ First, monomer electrodes with a relatively uniform dispersion are fabricated and assembled into batteries. During the charging process, the small‐molecule monomer electrode then electrochemically polymerizes inside of the batteries, forming a poorly soluble polymer electrode. However, because it is also difficult for monomer particles to achieve nano‐sized dispersion in the composite electrode, the in situ formed polymer particles will also be relatively large, hindering further electrochemical performance enhancement.^[^ ^36^, ^38^ ^]^

Compositing small‐molecule electrode materials with porous carbon materials are another important method to suppress dissolution.^[^ ^40^, ^41^, ^42^, ^43^ ^]^ In particular, owing to its well‐ordered porous structure, large specific surface area, high conductivity, and, importantly, its nano‐dispersion/confinement effect,^[^ ^44^ ^]^ ordered mesoporous carbon (CMK‐3) has been frequently employed to load soluble organic small molecules and alleviate their dissolution (Scheme 1b), achieving improved cycle performance.^[^ ^41^, ^42^, ^43^, ^44^, ^45^, ^46^, ^47^ ^]^ Additionally, when organic molecules are dispersed in the highly‐ordered nanopores of CMK‐3, CMK‐3 can provide abundantly conductive pathways, which dramatically facilitates ion/electron diffusion and transport, thus ensuring rapid reaction kinetics.^[^ ^44^ ^]^ However, because of the inevitable dissolution of organic small molecules from the pores of CMK‐3 after long durations, these reported organic electrodes still underwent a slow capacity decay with cycling ability of no <500 cycles.^[^ ^42^, ^44^ ^]^ Therefore, to further improve the electrochemical performance, it is highly desirable to develop a more effective method to simultaneously achieve long‐term dissolution inhibition and electrode architecture optimization.

This study proposes a new strategy for in situ electropolymerization in the nanopores of CMK‐3, as shown in Scheme 1c, to solve the above‐mentioned problems by combining the advantages of both mesoporous carbon and the in situ electropolymerization method. After electropolymerization in CMK‐3, the formed polymers are then very difficult to dissolve even after a long duration, which is favorable for achieving a long cycle life. More importantly, as monomers are dispersed at nano scale in the pores of CMK‐3, the formed polymers must also be nano‐sized. The nano‐sized polymer, together with the advantages of CMK‐3 (that is, well‐ordered porous structure, large specific surface area, and high conductivity) will help to achieve high utilization of active sites and electron/ion diffusion kinetics,^[^ ^48^ ^]^ leading to high capacity (close to its theoretical value), superb rate performance, and excellent low‐temperature performance. In this study, 1‐naphthylamine (NA), which can be polymerized under an electric field, was used to verify our hypothesis. Fourier transform infra‐red (FTIR) spectra, UV–vis spectra, and scanning electron microscopy (SEM) characterizations were used to confirm the formation of nanostructured poly(1‐naphthylamine) (PNA). The obtained NA/CMK‐3 cathode delivers a high active site utilization of 93.7% (175.4 mAh g^−1^) at 0.2 A g^−1^, ultrafast rate performance of up to 60 A g^−1^, and ultralong cycle life of 10 000 cycles at room temperature and 45 000 cycles at a low temperature of −15 °C. These results demonstrate that in situ electropolymerization in porous carbon is an effective method for enhancing the electrochemical performance of organic electrode materials with long cycle life and excellent kinetics performance.

## 2 Results and Discussion

### 2.1 Characterization of NA/CMK‐3 Nanocomposites

**Figure 1:**
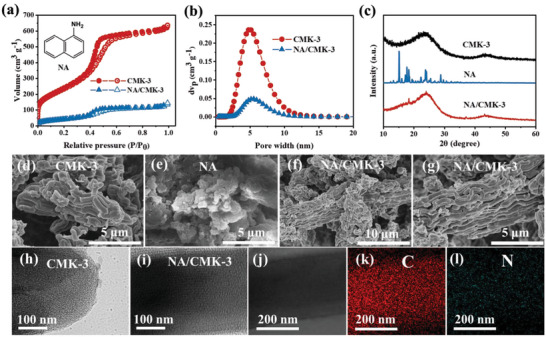
Characterization of the NA/CMK‐3 nanocomposites. a) Nitrogen adsorption/desorption isotherms and b) corresponding PSD profiles of the CMK‐3 and NA/CMK‐3 nanocomposites. c) XRD patterns of the CMK‐3, NA, and NA/CMK‐3 nanocomposites. SEM images of the d) CMK‐3, e) NA, and f,g) NA/CMK‐3 nanocomposites. TEM images of the h) CMK‐3 and i) NA/CMK‐3 nanocomposites. j–l) TEM‐mapping images of the NA/CMK‐3 nanocomposites.

NA/CMK‐3 nanocomposites were prepared using a facile impregnation method (Figure S1, Supporting Information), and the details are provided in the experimental section of the Supporting Information. **Figure** **1**a,b show the nitrogen adsorption/desorption isotherms and corresponding pore size distribution (PSD) curves of CMK‐3 and the resulting nanocomposites, respectively. After encapsulating NA into CMK‐3, the Brunauer–Emmett–Teller surface area decreased from 868.7 to 156.1 m^2^ g^−1^. Meanwhile, the pore volume decreased from 1.07 to 0.21 cm^3^ g^−1^. Figure 1c shows the X‐ray diffraction (XRD) patterns of the CMK‐3, NA, and NA/CMK‐3 nanocomposites. The diffraction peaks of NA in the NA/CMK‐3 nanocomposites almost completely disappeared, implying that NA was loaded into the CMK‐3 nanopores.

Microstructures of the CMK‐3, NA, and NA/CMK‐3 nanocomposites were further characterized using SEM and transmission electron microscopy (TEM). SEM images (Figure 1d,e) show that CMK‐3 and NA had rod‐ and block‐like particles, respectively. After impregnation, the NA/CMK‐3 nanocomposites (Figure 1f,g) exhibited a uniform morphology, and almost no NA could be observed. TEM images of the CMK‐3 and NA/CMK‐3 nanocomposites are shown in Figure 1h,i, respectively. The nanopores of NA/CMK‐3, as compared to those of pristine CMK‐3 (Figure 1h), were visibly filled. The elemental compositions of the CMK‐3 and NA/CMK‐3 nanocomposites were also analyzed by TEM mapping (Figure 1i,j; Figure S2, Supporting Information), which clearly indicated that carbon and nitrogen were homogeneously distributed in the NA/CMK‐3 nanocomposites. The above results strongly demonstrate that NA can be successfully encapsulated into the nanopores of CMK‐3 with uniform dispersion using the facile impregnation method.

For comparison, PNA was also synthesized ex situ using chemical oxidative polymerization of NA in the presence of FeCl_3_ under anhydrous and oxygen‐free conditions (denoted as ex‐PNA). The synthetic route is shown in Figure S3 (Supporting Information) and the chemical structure was confirmed by using FTIR (Figure S4, Supporting Information). In contrast, the SEM characterizations (Figure S5, Supporting Information) clearly show that ex‐PNA cannot enter the nanopores of CMK‐3 because of their large particle size.

### 2.2 Electrochemical Performance of the NA/CMK‐3 Cathode

**Figure 2:**
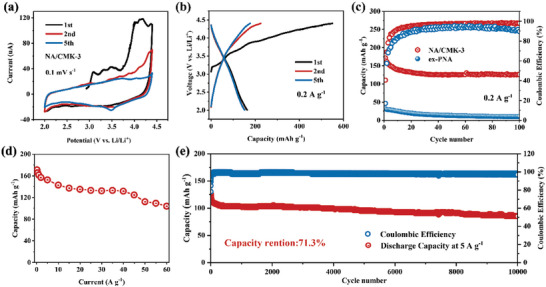
Electrochemical performance of the NA/CMK‐3 cathodes. a) CV curves of the NA/CMK‐3 cathodes at 0.1 mV s^−1^ in the voltage range of 2.0–4.4 V. b) Galvanostatic charge/discharge profiles of the NA/CMK‐3 cathode at 0.2 A g^−1^. c) Cycle performance of the NA/CMK‐3 and ex‐PNA cathodes. d) Rate performance of the NA/CMK‐3 cathode at various current densities. e) Long‐term cycle performance of the NA/CMK‐3 cathode cycled at 2.0–4.2 V at 5 A g^−1^.

Electrochemical performance of the NA, ex‐PNA, and NA/CMK‐3 cathodes were evaluated in coin‐type cells using lithium foil as the anode. Cyclic voltammetry (CV) tests were performed at a scan rate of 0.1 mV s^−1^ in the voltage range of 2.0–4.4 V versus Li/Li^+^ (**Figure** **2**a). In the first anodic scan, the strong peak at ≈4.1 V was attributed to the electrochemical polymerization of NA (vide post), whereas the small peaks at 3.1–3.7 V were ascribed to the oxidation reaction of amino group and side reactions.^[^ ^49^, ^50^ ^]^ The significant difference in the cycle performance of the NA/CMK‐3 cathode at 2.0–3.6 and at 2.0–4.1 V demonstrates that the peak at ≈4.1 V can be ascribed to electrochemical oxidation polymerization (vide post) (Figure S6, Supporting Information). The oxidation peaks at 3.1–3.7 V disappeared after the first cycle and were replaced by a broad peak, indicating the formation of polymer after the first charge.^[^ ^36^, ^37^, ^38^ ^]^ Galvanostatic charge/discharge profiles of the NA/CMK‐3 cathode were consistent with the CV results (Figure 2b). In the first charging process, after the appearance of several short voltage plateaus at 3.2–3.8 V, a long sloping voltage plateau was observed from ≈4.1 V. Thereafter, sloping voltage profiles without an obvious plateau were presented in subsequent cycles with good reversibility.

As shown in Figure 2c, the electrochemical performance of NA/CMK‐3 electrode, as compared to that of the conventional polymer electrode (ex‐PNA), was significantly enhanced. The first discharge capacity of the NA/CMK‐3 cathode was 175.4 mAh g^−1^ (C_th_ = 187.2 mAh g^−1^) after removing the capacity contribution of CMK‐3, corresponding to a high active site utilization of 93.7% (Figures S7–S9, Supporting Information). In contrast, the ex‐PNA cathode only delivered a capacity of 32.8 mAh g^−1^, corresponding to a very low active site utilization of 17.5% (Figure 2c; Figure S10, Supporting Information). For comparison, the electrochemical performance of the NA electrode without CMK‐3 encapsulation was also studied. However, it exhibited a very low initial discharge capacity of 11.5 mAh g^−1^ (Figure S11, Supporting Information) because of severe dissolution.^[^ ^38^ ^]^

The significant performance improvement of the NA/CMK‐3 electrode is clearly related to the use of CMK‐3. Because NA is extremely soluble in the electrolyte, if NA is not loaded in CMK‐3, it will be largely dissolved instead of polymerized, resulting in a low capacity. For the NA/CMK‐3 electrode, CMK‐3 could effectively inhibit the dissolution of NA during the early stage because of the nano‐confinement effect (Figure S12, Supporting Information) and thus, NA could be successfully in situ electropolymerized, resulting in high capacity and enhanced cycling stability. Moreover, the in situ formed PNA in CMK‐3 was nano‐sized owing to its nano‐dispersion effect, as compared to the ex‐PNA electrode with large polymer particles. The large particle size of ex‐PNA buried many active sites, whereas nano‐sized PNA in CMK‐3 exposed more active sites (that is, higher active site utilization, and thus also high capacity).^[^ ^51^ ^]^ These results demonstrate that the severe dissolution of small‐molecule electrode materials and agglomeration of polymer electrode materials can be efficiently and simultaneously solved by in situ electropolymerization in the nanopores of CMK‐3.

Rate performance of the in situ electropolymerized NA/CMK‐3 cathode was investigated at different current densities of 0.2–60 A g^−1^ in a voltage range of 2.0–4.4 V (Figure 2d; Figure S13, Supporting Information). When the current density increased from 0.2 to 40 A g^−1^ (≈213 C), the reversible capacities only decreased from 175.4 to 131.5 mAh g^−1^. Even at an extremely high current density of 60 A g^−1^ (≈320 C), the NA/CMK‐3 cathode could still deliver a high capacity of 118.6 mAh g^−1^, corresponding to a high capacity retention of 67.6%. Figure S14 (Supporting Information) summarizes the Ragone plots of the NA/CMK‐3 cathode, as compared to state‐of‐the‐art organic cathode materials. Remarkably, the NA/CMK‐3 cathode delivered a maximum energy density of 312 Wh Kg^−1^ and a maximum power density of 161364 KW Kg^−1^. The rate capability outperforms all previously reported organic cathode materials for LIBs.

Its long‐term cycle performance was also investigated. As a high cutoff voltage will inevitably cause electrolyte decomposition,^[^ ^36^, ^38^ ^]^ the long‐term cycling performance of NA/CMK‐3 was evaluated in the voltage range of 2.0–4.2 V at a high current density of 5 A g^−1^ (Figure 2e). NA/CMK‐3 exhibited excellent cycling stability, with a high‐capacity retention of 71.3% after 10 000 cycles at room temperature, which is among the best long‐term cycle performances reported thus far (Table S1, Supporting Information). This result demonstrates the huge advantage of our novel strategy, that is, the encapsulation of monomers within CMK‐3 and in situ electropolymerization, which offers a significant cycling stability enhancement.

### 2.3 Characterization of In Situ Electropolymerization

**Figure 3:**
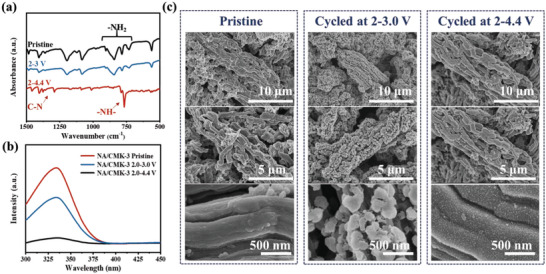
Characterization of the in situ electropolymerization. a) FTIR and b) UV–vis spectra, and c) SEM images of the pristine NA/CMK‐3 electrode and the NA/CMK‐3 cathode cycled at 2.0–3.0 and 2.0–4.4 V at 0.2 A g^−1^ after 5 cycles.

NA/CMK‐3 cathode cycled at 4.1 V displays better cycle performance with a capacity retention of 92.3% after 50 cycles at 0.2 A g^−1^, which is much higher than that of cycled at 3.0 V, 49.7% (Figure S15, Supporting Information). The poor cycling stability of NA/CMK‐3 cathode cycled at 2.0–3.0 V is definitely due to the dissolution of unpolymerized NA. Apparently, in situ electropolymerization can indeed effectively improve the cycle performance of NA/CMK‐3 cathode. To further confirm the electropolymerization of the NA/CMK‐3 cathode during cycling, FTIR tests were first conducted on the pristine and fully discharged NA/CMK‐3 cathodes at different cutoff voltages (**Figure** **3**a). When the electrode was cycled at 2.0–3.0 V, no changes were observed. In contrast, when the electrode was cycled at 2.0–4.4 V, the broad and strong peaks at 900–700 cm^−1^, which were assigned to the out‐of‐plane deformation vibration of −NH_2_ of NA, became a narrow peak of —NH— at 766 cm^−1^ after polymerization. Moreover, a new peak appeared at 1288 cm^−1^, which was ascribed to the stretching vibration absorption of the newly formed carbon‐nitrogen bond.^[^ ^28^ ^]^ Furthermore, the UV–vis spectra of soaked 1,2‐dimethoxythane solutions of pristine and cycled cathodes were compared to examine the solubility change of the active materials before and after polymerization. Figure 3b shows that both the pristine electrode and electrode cycled at 2.0–3.0 V exhibited strong absorption peaks, indicating severe dissolution of NA. In contrast, almost no absorption signal was observed for the electrode cycled at 2.0–4.4 V, further suggesting the formation of insoluble polymers at high voltages.

To explore the effect of CMK‐3, the morphologies of the pristine and cycled NA/CMK‐3 electrodes were further examined by SEM (Figure 3c). Compared with the pristine electrode, the active materials appeared on the surface of CMK‐3 after cycling at 2.0–3.0 V, confirming the dissolution of NA from the nanopores of CMK‐3 during electrochemical cycling. In sharp contrast, nanostructured PNA was clearly observed on the surface of CMK‐3 when cycled at 2.0–4.4 V, demonstrating that electrochemical oxidation polymerization of NA occurred in the nanopores of CMK‐3, and the formed polymers thereafter is difficult to dissolve from the inside of CMK‐3. The morphologies of NA/CMK‐3 electrodes after cycled for 100 times were further investigated (Figure S16, Supporting Information) and it is also found that polymers cannot dissolve out of CMK‐3, demonstrating the superior stability of NA/CMK‐3 electrode. All of the above results provide compelling evidence that in situ electropolymerization indeed occurred in CMK‐3, yielding outstanding cycling stability.

### 2.4 Study on the Kinetics and Low‐Temperature Properties of the NA/CMK‐3 Cathode

**Figure 4:**
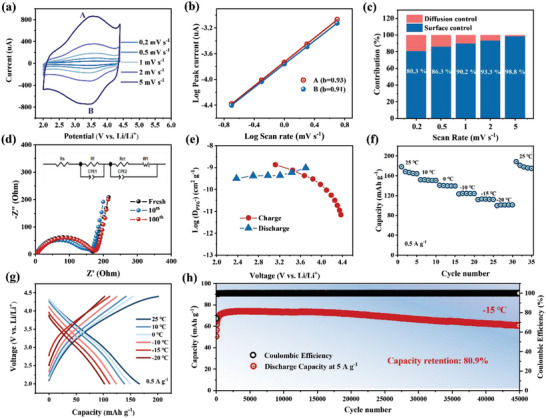
Kinetics and low temperature properties of the NA/CMK‐3 cathode. a) CV curves of the NA/CMK‐3 cathode at various scan rates (A and B represent the cathodic and anodic peaks, respectively). b) Liner fitting results of the logarithm (log) relationship between peak current and scan rate for the NA/CMK‐3 cathode. c) Capacity contribution ratios of diffusion control (orange) and surface control (blue) at different scan rates. d) Impedance evolution with cycling of the NA/CMK‐3 cathode. e) Diffusion coefficient of PF_6_^−^ calculated from the GITT curves. f) Discharge capacity of NA/CMK‐3 at 0.5 A g^−1^ at different temperatures and g) their corresponding galvanostatic charge/discharge profiles. h) Long‐term cycle performance under 5 A g^−1^ at −15 °C.

The excellent rate performance reported above suggests fast ion/electron diffusion. Therefore, the reaction kinetics of the NA/CMK‐3 electrode was further explored. CV measurements were performed for both the NA/CMK‐3 and ex‐PNA cathodes at the voltage scan rates of 0.2–5 mV s^−1^ (**Figure** **4**a; Figure S17, Supporting Information). The total capacity generally consists of two parts, namely, a faradaic and a capacitive process, which can be analyzed by the relationship between the peak current (*i*) and voltage scan rate (*v*) according to the formula *i = a v^b^*, where *a* and *b* are positive variables. A *b* value close to 0.5 represents a Faradaic process, whereas for a capacitive process, this value is close to 1.0.^[^ ^52^, ^53^ ^]^ The *b* values from the cathodic and anodic peaks of the NA/CMK‐3 electrode, as shown in Figure 4b, was calculated to be 0.93 for peak A and 0.91 for peak B, indicating that its capacity is mainly controlled by a capacitive contribution. Even at a low scan rate of 0.2 mV s^−1^, 80.3% of the capacity still originated from the capacitive process (Figure 4c; Figure S18, Supporting Information). In strike contrast, only 31.4% of the capacity of the ex‐PNA electrode originated from the capacitive process at 0.2 mV s^−1^ (Figures S19 and S20, Supporting Information), indicating poor kinetics.

To further probe the reaction kinetics, in situ electrochemical impedance spectroscopy (EIS) measurements were conducted for the NA/CMK‐3 and ex‐PNA cathodes (Figure 4d; Figure S21, Supporting Information). Nyquist plots were fitted with equivalent electric circuits (insets in Figure 4d; Figure S21, Supporting Information), and the electrolyte resistance (*R*_s_), interfacial resistance (*R_f_*), and charge transfer resistance (*R_ct_*) are compared in Table S2 (Supporting Information). The NA/CMK‐3 cathode exhibited a stable and smaller *R_ct_*, whereas the ex‐PNA cathode exhibited a larger *R_ct_* that rapidly increased.

The diffusion coefficient (*D*) of the NA/CMK‐3 electrode was examined to determine its ion‐transport properties. *D* can be quantitatively determined using $D\;=\frac{4}{\pi \tau }\;{\left(\frac{m_{B}V_{M}}{M_{B}S}\right)}^{2}{\left(\frac{\Delta E_{S}}{\Delta E_{\tau }}\right)}^{2}$, where *τ* is the pulse duration, *m*_B_ and *M*_B_ are the mass and molar mass of the active materials, respectively, *V*_M_ is the molar volume, and *S* is the active surface area of the cathode. ∆*E*_s_ and ∆*E*_*τ*_ were obtained from the GITT curves (Figure S22, Supporting Information). As depicted in Figure 4e, the calculated *D* values were 0.3 × 10^−8^ to 0.4 × 10^−10^ cm^2^ s^−1^, and the diffusion coefficients during the discharge process were at a high level of 10^−9^ cm^2^ s^−1^, which explains its excellent rate performance.^[^ ^54^, ^55^ ^]^ These results demonstrate that the in situ electropolymerized NA/CMK‐3 electrode exhibits excellent reaction kinetics.

It is known that electrode architecture plays a significant role in enhancing the reaction kinetics of composite electrodes.^[^ ^56^ ^]^ CMK‐3 cannot only control the in situ synthesized polymer at the nano scale but is also conducive to the construction of nanostructured electrodes. The porous NA/CMK‐3 electrode with large specific surface area and well‐ordered porous structure favors fast ion/electron transport, achieving a significantly enhanced rate capability.

Excellent low‐temperature performance is very important for LIBs and can extend their application to extreme conditions, such as in deserts and polar regions.^[^ ^20^, ^57^, ^58^ ^]^ The admirable kinetics properties of the NA/CMK‐3 electrode prompted its low‐temperature performance to be further investigated. As shown in Figure 4f,g, when tested at 25, 10, 0, ‐10, ‐15, and ‐20 °C, the NA/CMK‐3 electrode delivered reversible specific capacities of 168.1, 151.9, 140.9, 124.5, 113.1, and 101.2 mAh g^−1^ at 0.5 A g^−1^, respectively. The capacities at 10, 0, ‐10, ‐15, and ‐20 °C corresponded to 90.4, 83.8, 74.1, 67.3%, and 60.2% of those at room temperature, respectively, indicating superior low‐temperature performance. The cycle performance at a low temperature of −15 °C was then evaluated. Very high capacity retention of 95.6% was delivered after 100 cycles at current density of 0.2 A g^−1^ (Figure S23, Supporting Information). The NA/CMK‐3 electrode also exhibited excellent long‐term cycling stability with a high capacity retention of 80.9% after 45000 cycles at 5 A g^−1^ (Figure 4h). This result surpasses all previous results for organic/inorganic cathodes in LIBs or even zinc‐ion batteries reported in the literature (Table S3, Supporting Information).

### 2.5 Storage Mechanism

**Figure 5:**
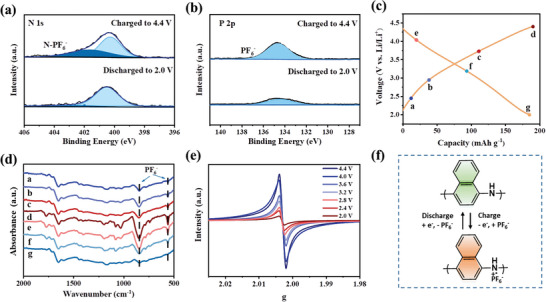
Storage mechanism of the in situ synthesized PNA. High‐resolution a) N 1s and b) P 2p of the NA/CMK‐3 cathode at 2.0 and 4.4 V, respectively. c) Galvanostatic charge/discharge curves of the NA/CMK‐3 cathode at 0.2 A g^−1^. Corresponding d) FTIR and e) EPR spectra. f) The PF_6_^−^ storage mechanism.

The storage mechanism of the in situ synthesized PNA was studied by ex situ X‐ray photoelectron spectroscopy (XPS), electron paramagnetic resonance (EPR), and FTIR spectroscopy. The full survey XPS spectrum of the pristine NA/CMK‐3 cathode exhibits three peaks located at 284.8, 400.2, and 687.8 eV, corresponding to C 1s, N 1s, and F 1s, respectively (Figure S24, Supporting Information).^[^ ^36^, ^59^ ^]^ The small peak associated with fluorine in the XPS spectrum of the pristine cathode was ascribed to poly(vinylidene fluoride) binder. Upon charging to 4.4 V, the fluorine content increased significantly, along with the emergence of two new peaks at 135 and 192 eV, corresponding to P 2p and P 2s, respectively, which were attributed to PF_6_^−^. After discharging to 2.0 V, the intensities of the P and F peaks decreased and the contents decreased accordingly, indicating the reversible extraction of PF_6_^−^ anions. This reversible process can be more clearly observed in the high‐resolution N 1s and P 2p (**Figure** **5**a,b; Figure S25, Supporting Information). The stronger 134.6 eV signal associated with PF_6_^−^ anions was detected in the charged state (4.4 V) with the appearance of a new N peak at 401.8 eV that was assigned to the N‐PF_6_^−^ interaction. However, the peak disappeared when discharged to 2.0 V, showing that anions can be completely extracted from the nitrogen atoms in oxidized PNA, thus demonstrating good reversibility.

To further explore the reaction mechanism, ex situ FTIR spectra were recorded at different charge/discharge states (marked in Figure 5c,d shows that the anionic PF_6_^−^ peak at 846.4 and 556.6 cm^−1^ became stronger with charging, which is indicative of the coordination of PF_6_^−^ with PNA, and became weaker during discharge and almost vanished at 2.0 V, indicating a reversible reaction between nitrogen atoms and PF_6_^−^ anions. Electron paramagnetic resonance (EPR) was also applied to the NA/CMK‐3 cathodes at different cutoff voltages (Figure 5e; Figure S26, Supporting Information). The intensity of the nitrogen radicals gradually strengthened with the charging process, indicating that more nitrogen atoms were transformed into nitrogen radicals. Based on the above results, the p‐type mechanism of the PNA is shown in Figure 5f. During the charging process, one ‐NH‐ group loses one electron, resulting in one‐electron oxidation of one NA unit. The positive charges are compensated by the uptake of PF_6_^−^ ions. During the discharge process, PF_6_^−^ extracts and neutral PNA regains.

## 3 Conclusion

In summary, a novel method for in situ electropolymerization in the nanopores of CMK‐3 was developed for the first time to fabricate nanostructured polymer cathodes for LIBs. Nano‐sized polymers can be effectively synthesized because of the nano‐dispersion and nano‐confinement effects of CMK‐3, which not only solves the dissolution problem but also improves the active site utilization. Moreover, the nanostructured polymer/mesoporous carbon electrode exhibited significantly improved reaction kinetics. Consequently, excellent electrochemical performance was demonstrated with a high active site utilization of 93.7%, ultrafast rate capability of 60 A g^−1^ (320 C), and an ultralong cycle life at both room temperature (10 000 cycles) and at a low temperature of −15 °C (45000 cycles). These results demonstrate that in situ electropolymerization in mesoporous carbon is a promising method for constructing high‐performance polymer electrodes for rechargeable lithium‐organic batteries.

## Conflict of Interest

The authors declare no conflict of interest.

## Supporting information

Supporting Information

## Data Availability

Research data are not shared.
