# Supplementary material for: High‐Performance Poly(1‐naphthylamine)/Mesoporous Carbon Cathode for Lithium‐Ion Batteries with Ultralong Cycle Life of 45000 Cycles at ‐15 °C
Source: Adv Sci (Weinh). 2023 Jun 9;10(23):2302490. doi: 10.1002/advs.202302490 (PMC10427393; doi:10.1002/advs.202302490)
Supplement: Supplementary file 1 — Supporting Information [file ADVS-10-2302490-s001.pdf]

ADVANCED SCIENCE
Open Access

Supporting Information

for *Adv. Sci.*, DOI 10.1002/advs.202302490

High-Performance Poly(1-naphthylamine)/Mesoporous Carbon Cathode for Lithium-Ion Batteries with Ultralong Cycle Life of 45000 Cycles at -15 °C

Junkai Yang, Xiaoru Zhao, Jixing Yang, Yunhua Xu and Yuesheng Li*

Supporting Information

High-Performance Poly(1-naphthylamine)/Mesoporous Carbon Cathode for Lithium-Ion Batteries with Ultralong Cycle Life of 45000 Cycles at -15 °C

Junkai Yang, Xiaoru Zhao, Jixing Yang, Yunhua Xu, and Yuesheng Li*

Experimental Section

Materials. All commercially available chemical raw materials, used without further purification, are listed below: 1-Naphthylamine (99% Adamas), CMK-3 (Nanjing JiCang co., China), Dichloromethane (DCM) was obtained from Tianjin Fuyu Fine Chemical Co., Lt dj.

Synthesis of Poly(1-naphthylamine) (ex-PNA). Under nitrogen atmosphere, 1-naphthylamine (143 mg 1 mmol) in 20 ml $CHCl_3$ was added dropwise into a suspension of $FeCl_3$ (486 mg 3 mmol) in 20 mL anhydrus $CHCl_3$. The mixture was stirred at room temperature for 24 hours, and then 100 mL methanol was added. The resulting precipitate was collected by filtration and washed by methanol for 3 times and 2 M hydrochloric acid solution, the product was collected and dry under vacuum for 7 hours to obtain purple poly(1-naphthylamine). Yield: 97 mg 68.8%.

Preparation of 1-Naphthylamine/CMK-3 Nanocomposites. The 1-naphthylamine/CMK-3 nanocomposites were prepared by a simple evaporation method. 1-naphthylamine was dissolved in the DCM and then ultrasonically treated for 10 minutes. After that, CMK-3 was added and the mixture was ultrasonically treated for an hour. The weight ratio of 1-Naphthylamine and CMK-3 was 1:2. Finally, the mixture was dried under vacuum to remove the solvent completely at room temperature for 6 hours.

Preparation of 1-Naphthylamine/CMK-3 Composite Electrode. The 1-naphthylamine/CMK-3 electrodes were prepared by mixing 1-naphthylamine/CMK-3 nanocomposites, carbon black and polyvinylidenefluoride (PVDF) in the ratio of 8:1:1 (wt%). Then moderate N-methylpyrrolidone (NMP) was added to form a well-dispersed slurry, the mixture was stirred for 1 hour and then casted onto a carbon-coating aluminum foil by a doctor blade. After drying at 60 °C for 3 hours, the electrode was punched into circular discs with a diameter of 9 mm.

Preparation of 1-Naphthylamine Electrode and Poly(1-Naphthylamine) Electrode. 1-Naphthylamine electrode and poly(1-naphthylamine) electrode were prepared by blending 1-Naphthylamine or poly(1-naphthylamine), carbon black and PVDF in a ratio of 6:3:1 (wt%). The mixture was grounded in a mortar for 1 hour. Then moderate NMP was added to obtain homogeneous slurry. The obtained mixture was casted on a carbon-coating aluminum foil by a doctor blade. After drying at 60 °C for 3 hours, the electrode was punched into circular discs with a diameter of 9 mm.

Cell Fabrication and Electrochemical Measurements. CR2032-type coin cells were assembled in argon-filled glovebox (O_2< 0.1 ppm, H_2O< 0.1 ppm) by the counter electrodes of lithium metal, polypropylene (PP) separators (Celgard 2500, LLC Corp., USA), and the electrolyte of 1 M $LiPF_6$ in ethylene carbonate: diethyl carbonate (EC: DEC) (1:1 by volume, 30 μL). The galvanostatic charge/discharge tests were performed on the Land test system (CT2001A, China). The electrochmical performace under different temperatures is carried out by putting the coin cells into a thermostat that can control temperature. Cyclic voltammograms at different scan rates between 2.0-4.4 V and Electrochemical Impedance Spectroscopy (EIS) spectra in the frequency of 10^5-0.01 Hz at 5 mV amplitude were recorded using Solartron 1470 Electrochemical Interface (Solartron Metrology, UK). In GITT measurements, a series of charge/discharge current pulses of 0.2 A g^{-1} with a duration of 5 min were applied followed by an open-circuit period of 1 h for each pulse.

Material Characterizations. The porous structures of the CMK-3 and 1-Naphthylamine/CMK-3 nanocomposites were analyzed by using N_2 (at 77 K) adsorption/desorption on a using a Bel Japan Inc. model BELSOPR-max analyzer. The samples were degassed at room temperature for 12 h under vacuum (10^{-5} bar) before the measurements. The pore size distribution was further calculated from the adsorption branch by nonlocal density functional theory (NLDFT) method. X-ray diffraction (XRD) patterns were collected on Rigaku Ultima IV through using Cu Kαradiation (λ=1.5406 Ȧ, 40KV and 40 mA). FTIR spectra were recorded by Bruker Alpha P spectrometer with reflection mode in emission from 4000 to 400 cm^{-1}. UV-vis spectra were measured on Shimadzu UV-3600 plus, Japan. The morphology and microstructures of samples were observed by Scanning Electron microscope (SEM, S-4800, Japan) and Transmission Electron microscope (TEM, JEM-2100F, Japan). X-ray photoelectron spectroscopy (XPS, K-Alpha, UK) was applied to analyze the chemical composition of the pristine and cycled electrodes. Electron paramagnetic resonance (EPR) test was performed on the JES-FA200 to monitor the strength of nitrogen radicals at

different voltages. The cycled electrodes were washed with diethyl carbonate (DEC) before measurements.

Figures and Tables

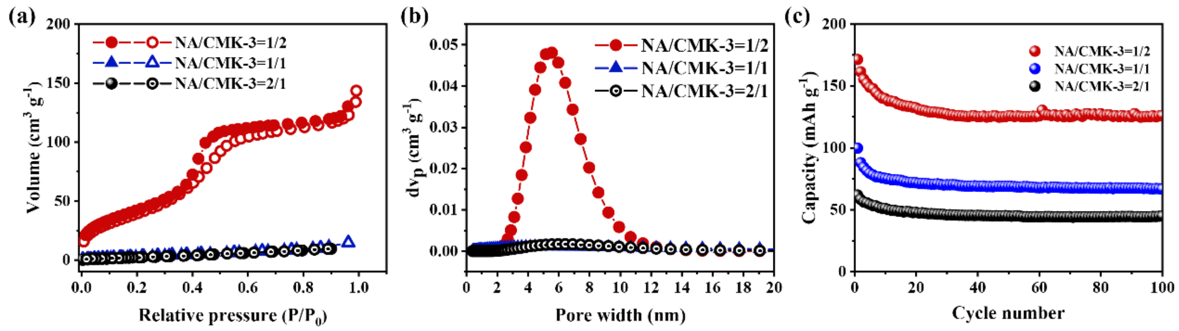

Figure S1. (a) Nitrogen adsorption/desorption isotherms and (b) corresponding pore size distribution profiles of NA/CMK-3=1/2, NA/CMK-3=1/1 and NA/CMK-3=2/1 Nanocomposites. (c) Cycle performance of NA/CMK-3=1/2, NA/CMK-3=1/1 and NA/CMK-3=2/1 electrodes.

The calculated Brunauer−Emmett−Teller specific surface area of NA/CMK-3=1/2, NA/CMK-3=1/1 and NA/CMK-3=2/1 are 156.1 m^2 g^{-1}, 15.73 m^2 g^{-1} and 15.13 m^2 g^{-1}, respectively (Figure S1a). Simultaneously, the pore volume also decreased accordingly, from 0.21 cm^3 g^{-1} of NA/CMK-3=1/2 to 0.02 cm^3 g^{-1} of NA/CMK-3=1/1 to 0.01 cm^3 g^{-1} of NA/CMK-3=2/1 (Figure S1b). These results indicate that NA/CMK-3 electrode with lower active materials content displays higher specific surface area and pore volume, which is beneficial to the diffusion and transport of large-sized anions (PF_6^-) and more utilization of active sites. To prove it, their electrochemical performance was also studied. As shown in Figure S1c, indeed, the electrode with low NA content shows higher capacity. Therefore, herein, we selected the ratio of NA/CMK-3=1/2 to prepare monomer/mesoporous carbon nanocomposites and thereafter composite electrodes to test the electrochemical performance.

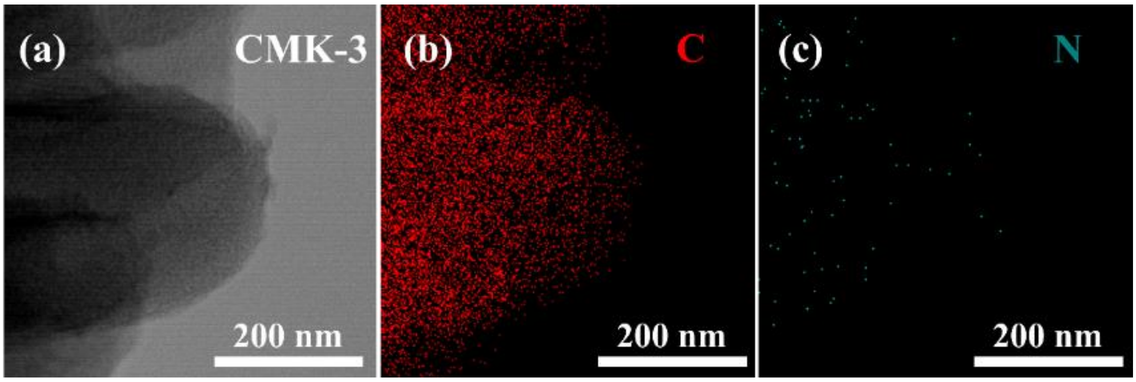

Figure S2. TEM images of (a) CMK-3 and (b, c) its TEM-mapping.

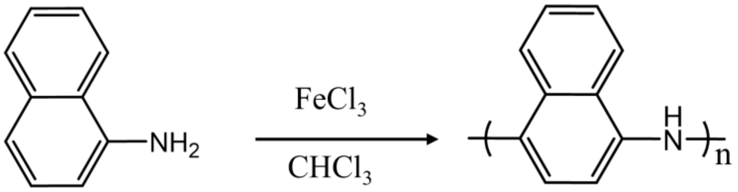

Figure S3. The synthetic route of ex-PNA.

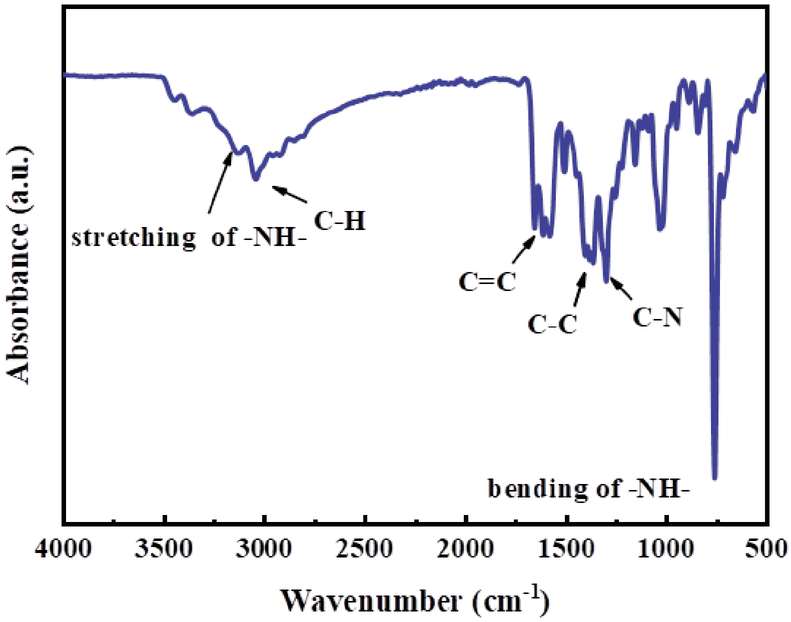

Figure S4. FTIR spectrum of ex-PNA.

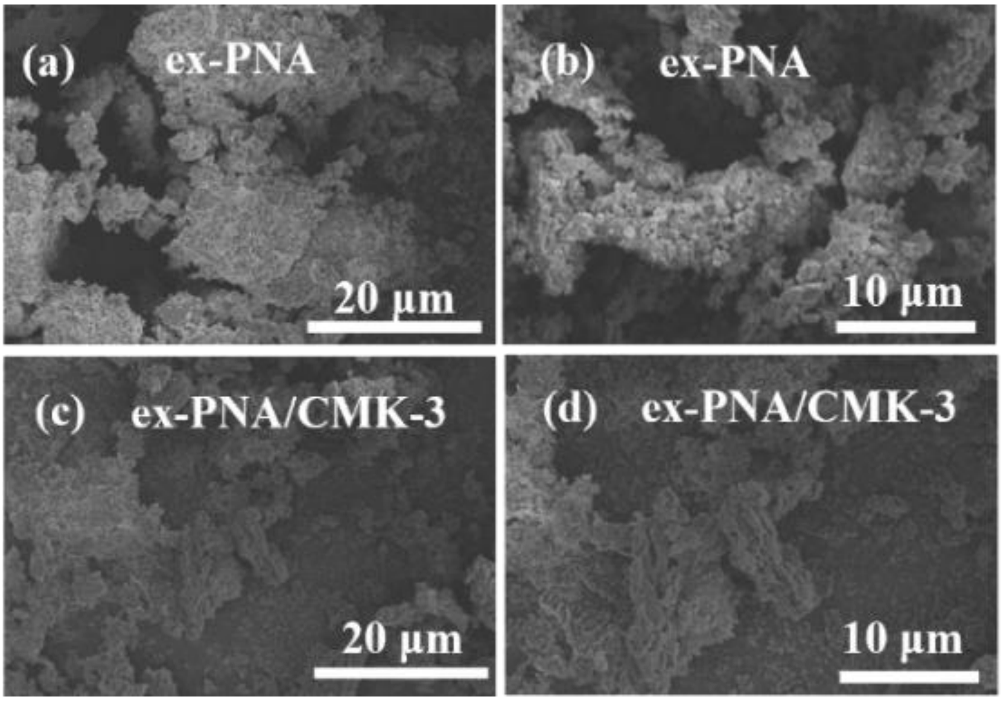

Figure S5. SEM images of (a,b) ex-PNA and (c,d) ex-PNA/CMK-3 nanocomposites.

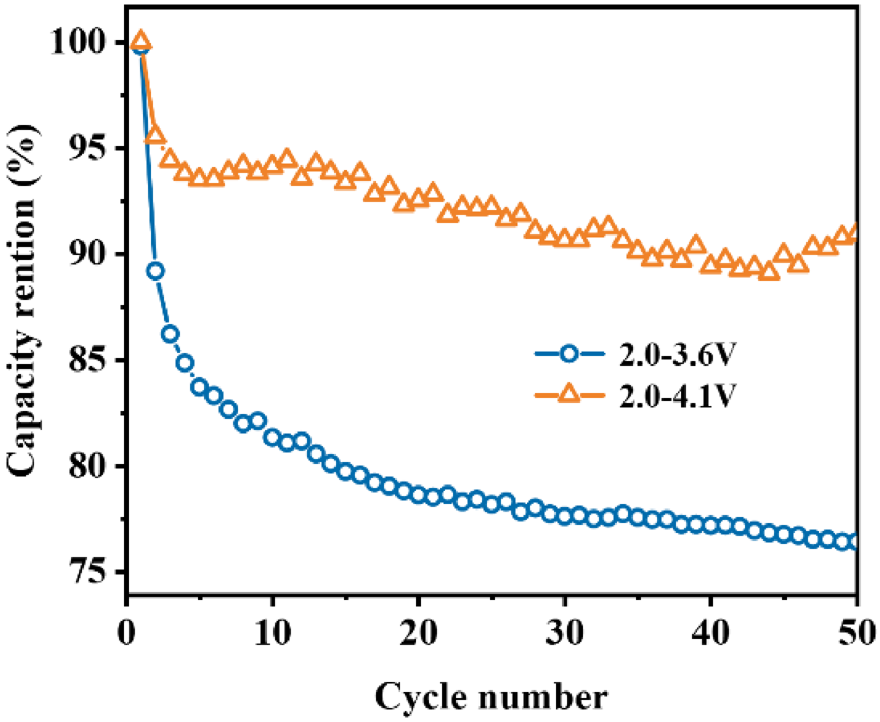

Figure S6. Cycling stability of NA/CMK-3 cathodes of different voltage ranges at 0.2 A g^{-1}.

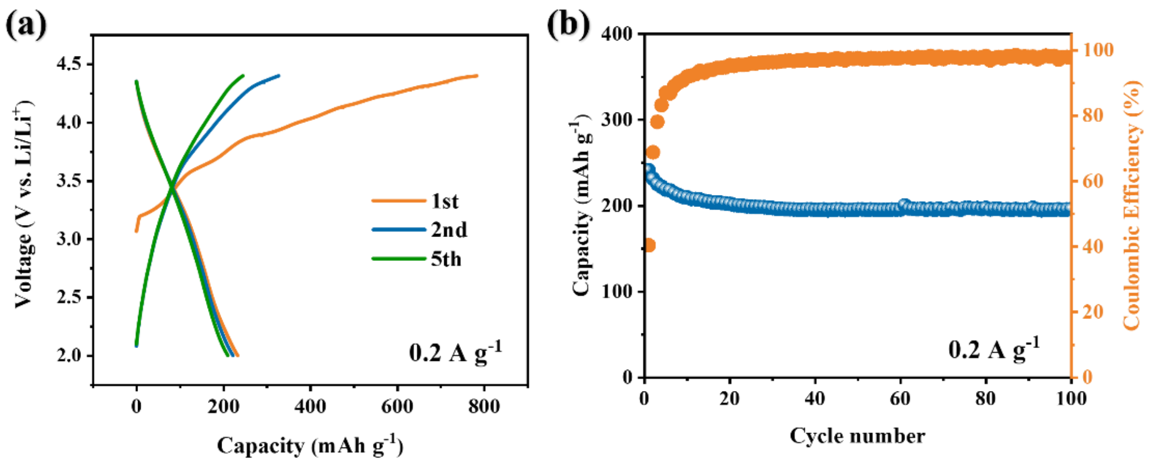

Figure S7. (a) The original galvanostatic charge/discharge (GCD) curves of NA/CMK-3 electrode and (b) corresponding cycle performance without removing the contributions of porous carbons.

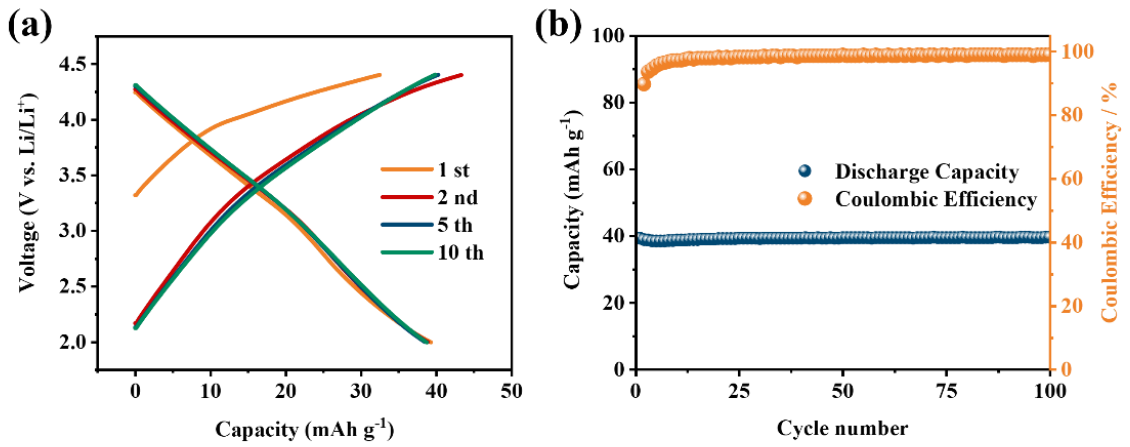

Figure S8. The galvanostatic charge/discharge curves and cycle performance of (a, b) CMK-3 (CMK-3: PVDF = 9:1) at 0.2 A g^{-1}.

The NA/CMK-3 cathodes contain 26.7 wt.% of active materials, 53.3 wt.% of CMK-3, 10 wt.% Super P and 10 wt.% PVDF. The capacity contribution of Super P can be neglected at voltage above 2.0 V. The capacities were based on the active materials and the capacity contribution of CMK-3 in the electrodes was subtracted. Figure S8 shows that capacity contributions of CMK-3, deliver ca. 40, respectively. The actual capacity of active materials was calculated using the following equation: $C_{\text{active material}} = C_{\text{measure}} - (53.3\%/26.7\%) \times C_{\text{CMK-3}}$

Where, $C_{\text{active material}}$, actual specific capacity of active materials (mAh g^{-1})

C_{measure} , measured capacity (mAh g^{-1})

$C_{\text{CMK-3}}$, measured capacity of CMK-3 or AC (mAh g^{-1})

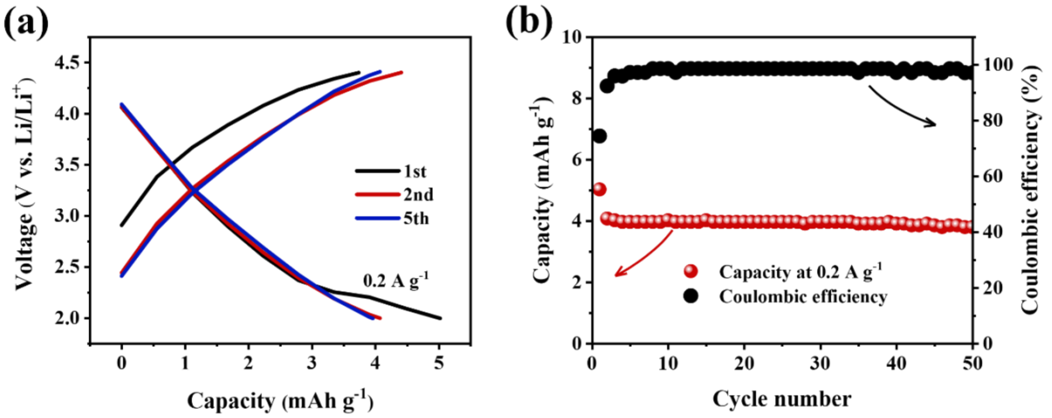

Figure S9. (a) The GCD curves and (b) corresponding cycle performance of super P cathode in the voltage range of 2.0-4.4 V**.**

We prepared and tested the electrochemical performance of the super P electrodes (super P: PVDF=9: 1) in the voltage range of 2.0-4.4 V. As shown in Figure S9, it only delivers about 4 mAh g^{-1} at current density of 0.2A g^{-1} in the voltage range of 2.0-4.4 V. According to the formula, $C_{Super\ P} = \frac{10\%}{26.7\%} \times C_{measure}$, the contribution of super P in the NA/CMK-3 electrodes is only about 1.5 mAh g^{-1}. Therefore, the contribution of super P can be omitted.

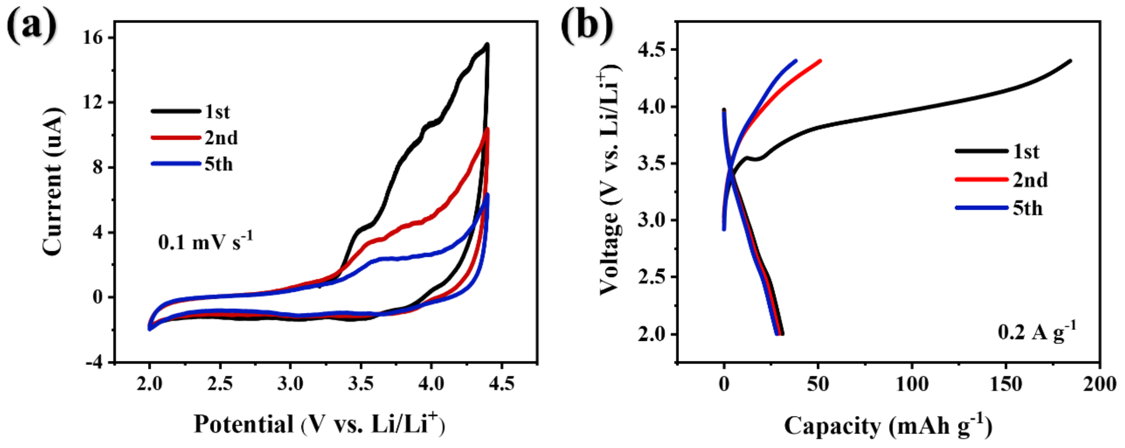

Figure S10. (a) CV curves at 0.1 mV s^{-1} and (b) GCD profiles of ex-PNA electrodes at 0.2 A g^{-1} in the voltage range of 2.0-4.4 V.

Although ex-PNA is a polymer, end units of polymer chains can also be polymerized under high-voltage condition. This phenomenon can be found in both our previous work (*ChemSusChem* 2021, 14, 4573-4582) and others' report (*Energy Storage Materials* 2022, 52, 465-472; *Chem. Commun.* 2020, 56, 5437-5440). However, compared with NA/CMK-3 cathode (Figure 2a), in situ electropolymerization in ex-PNA cathode was quite scarce as demonstrated by the very low current response of the polymerization peak of ex-PNA (Figure S10a). This is very reasonable because of the very few polymerizable groups in ex-PNA.

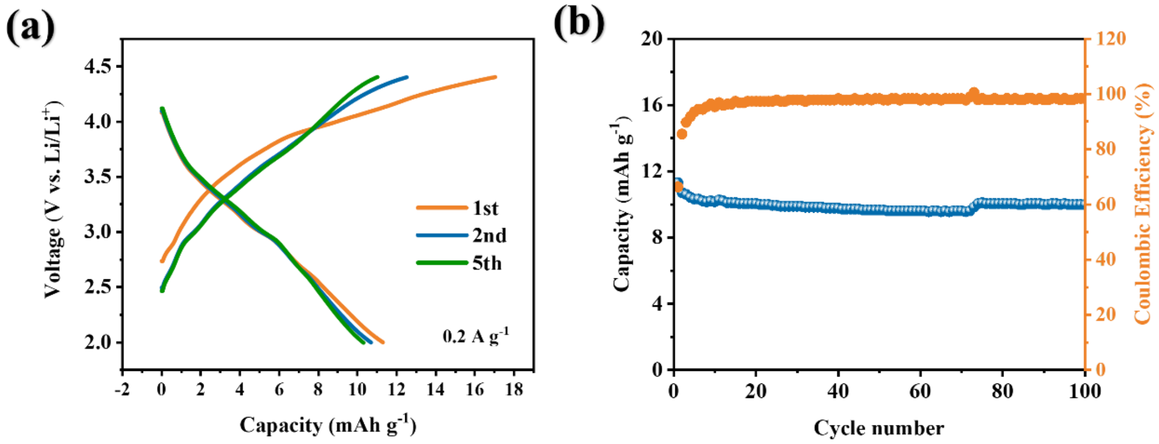

Figure S11. (a) The GCD profiles of NA cathode (without compositing with CMK-3) at 0.2 A g^{-1} and (b) corresponding cycle performance.

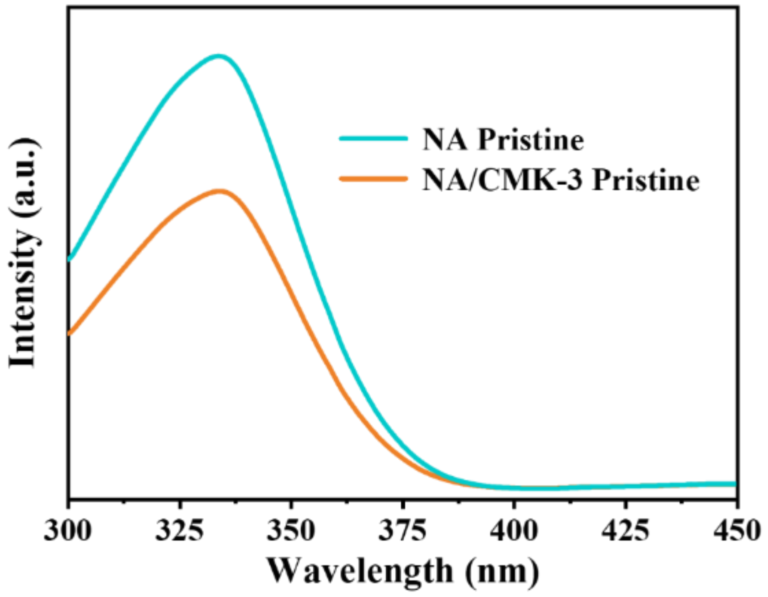

Figure S12. UV-vis spectra of the soaked 1,2-dimethoxythane solution of pristine NA and NA/CMK-3 cathodes for 12 hours.

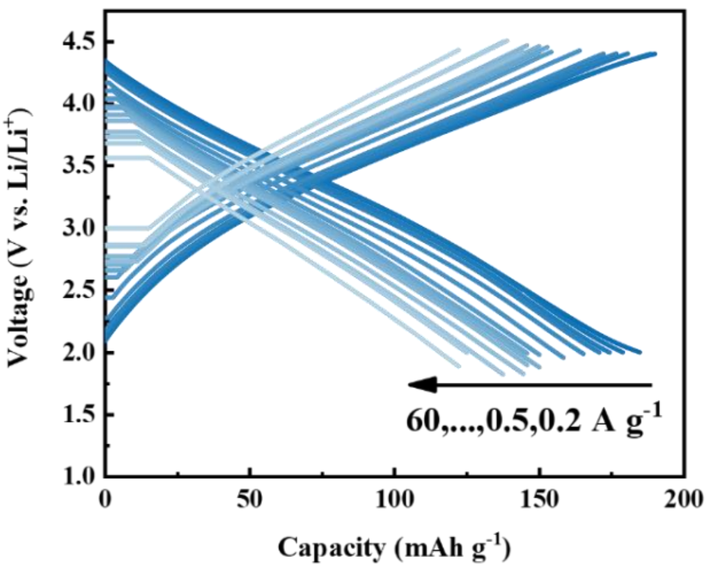

Figure S13. GCD profiles of NA/CMK-3 cathode at differnet current densities from 0.2 A g^{-1} to 60 A g^{-1}.

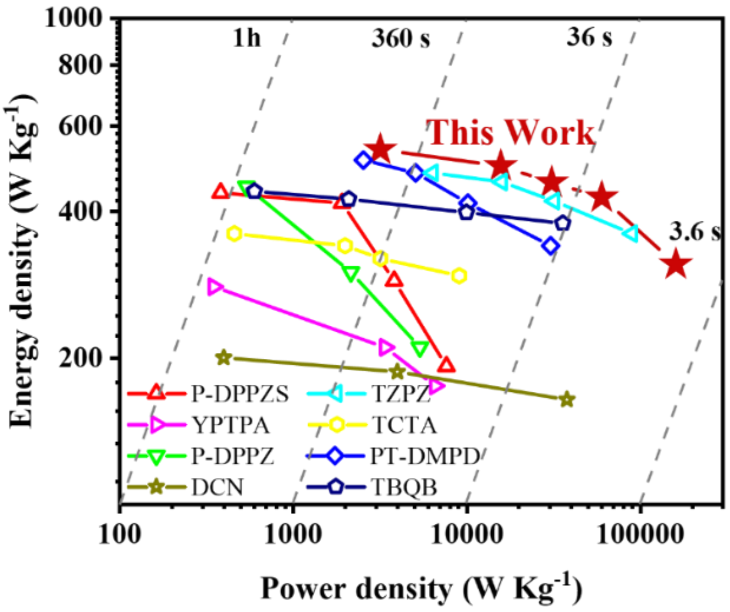

Figure S14. Ragone plots of NA/CMK-3 cathode in comparison with the state-of-the-art organic cathode materials for LIBs.

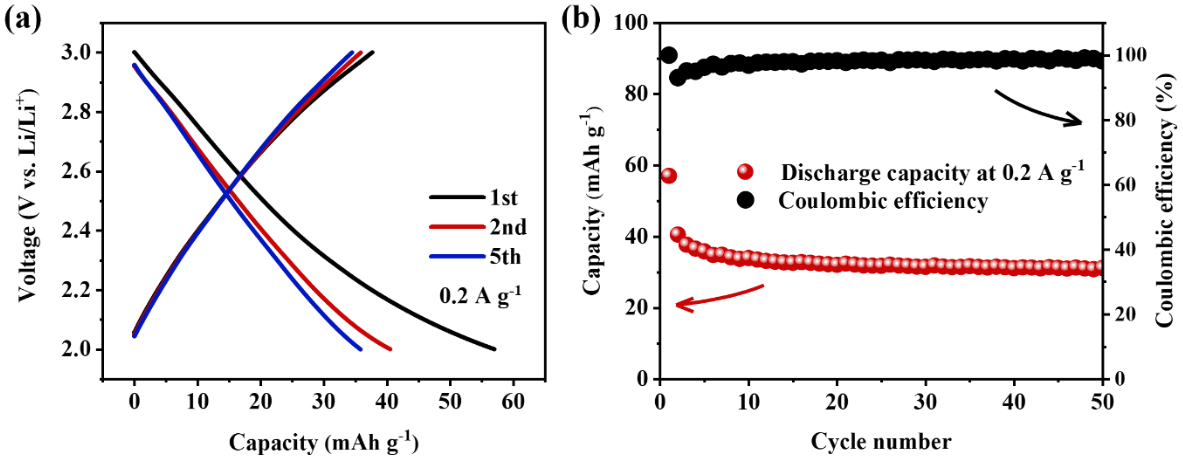

Figure S15. (a) The GCD profiles of NA/CMK-3 cathode at 0.2 A g^{-1} in the voltage range of 2.0-3.0 V and (b) corresponding cycle performance.

As no polymerization reaction happens in this voltage range, there is no obvious polymerization plateau in the first charging process and it displays greatly decreased capacity.

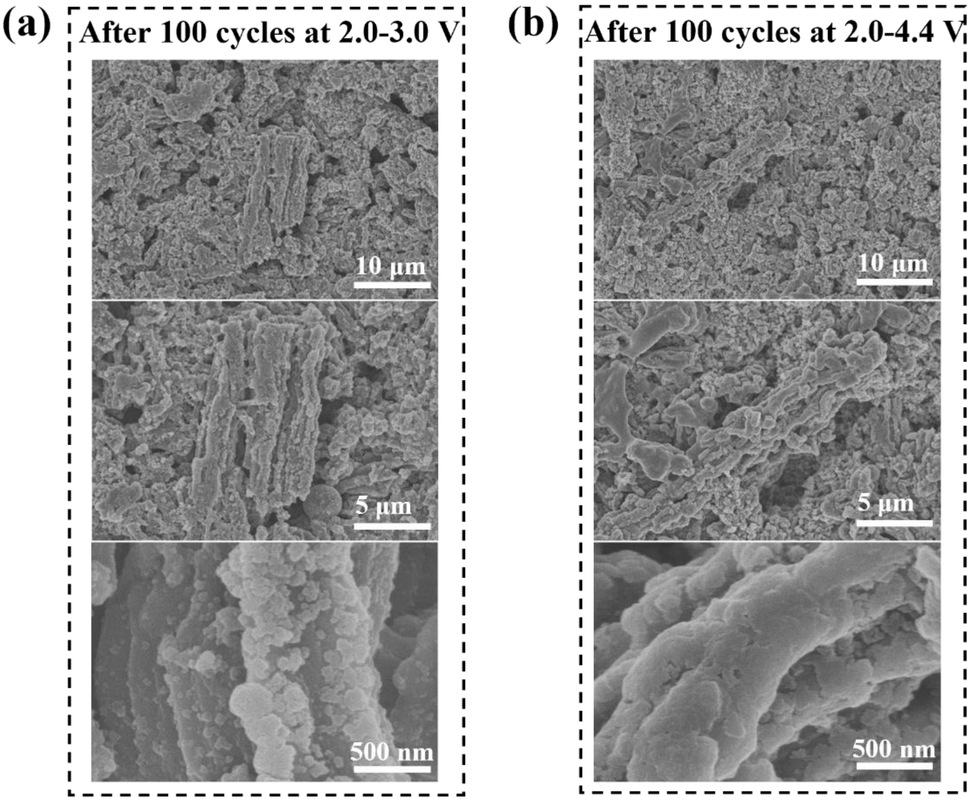

Figure S16. SEM images of NA/CMK-3 electrodes cycled in 2.0-3.0 V and 2.0-4.4 V at 0.2 A g^{-1} after 100 cycles.

In order to show the stability of NA/CMK-3 electrode in the liquid electrolyte, SEM images of NA/CMK-3 electrodes cycled in 2.0-3.0 V and 2.0-4.4 V at 0.2 A g^{-1} after long duration of 100 cycles were obtained and examined. As shown in Figure S16a, when cycled at 2.0-3.0 V, still, NA dissolved out of CMK-3 can be clearly observed on the surface of CMK-3. In contrast, when cycled at 2.0-4.4 V, as shown in Figure S16b, no NA particles could be found on the surface of CMK-3. These results further demonstrate the good stability of NA/CMK-3 electrode after in situ electropolymerization.

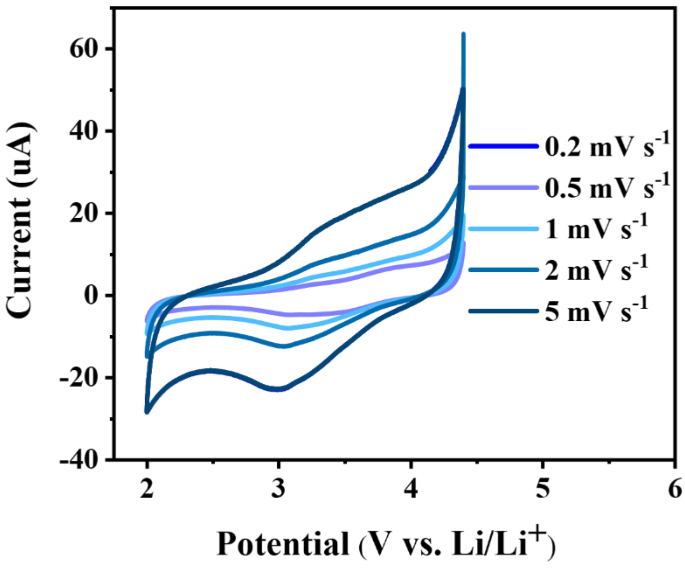

Figure S17. CV curves of ex-PNA cathode at various scan rates from 0.2 to 5 mV s^{-1}.

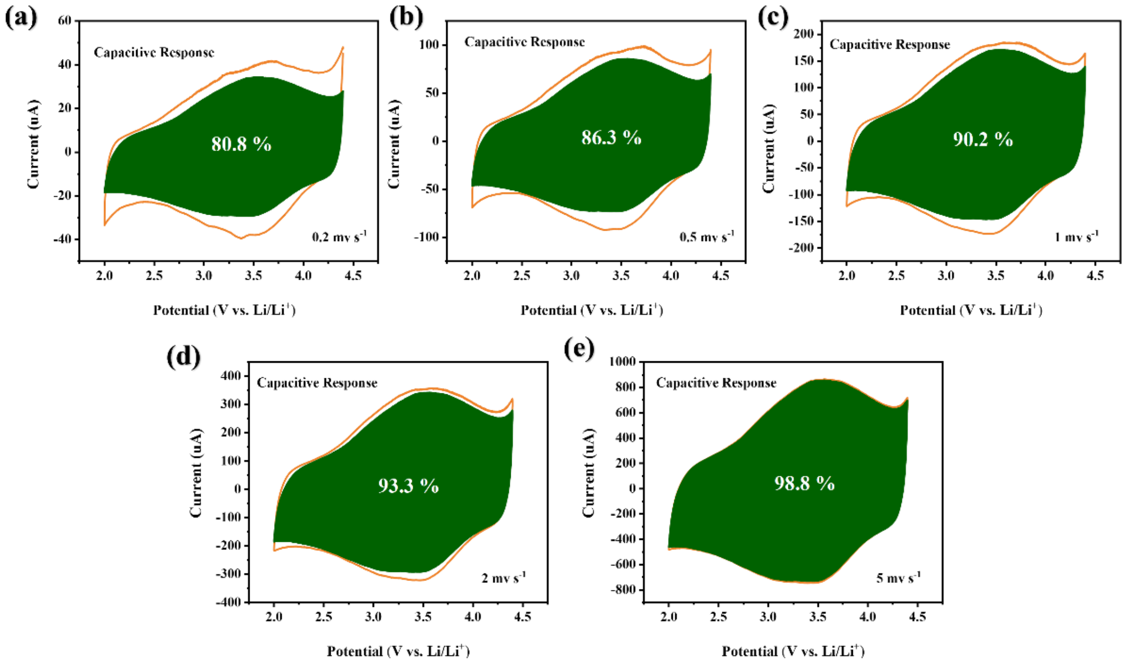

Figure S18. CV curves of NA/CMK-3 cathode and its corresponding capacitive contributions at different scan rates of (a) 0.2 mV s^{-1}, (b) 0.5 mV s^{-1}, (c) 1.0 mV s^{-1}, (d) 2.0 mV s^{-1}, (e) 5 mV s^{-1}.

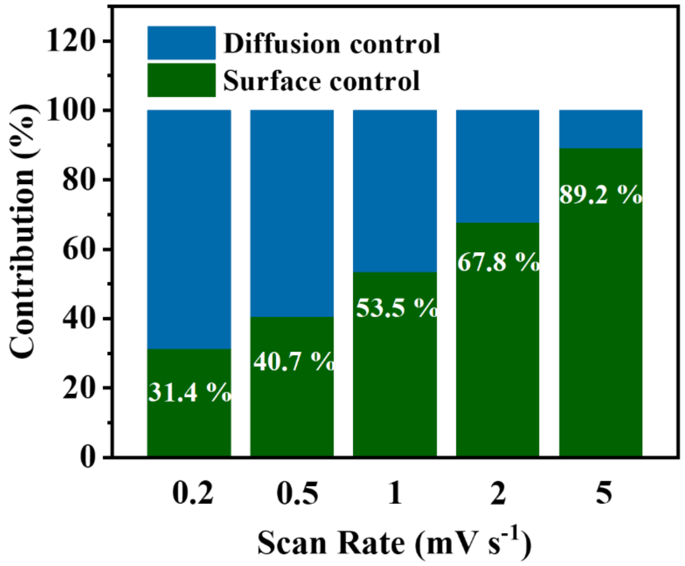

Figure S19. Capacity contribution ratios of diffusion control (blue) and surface control (green) at different scan rates.

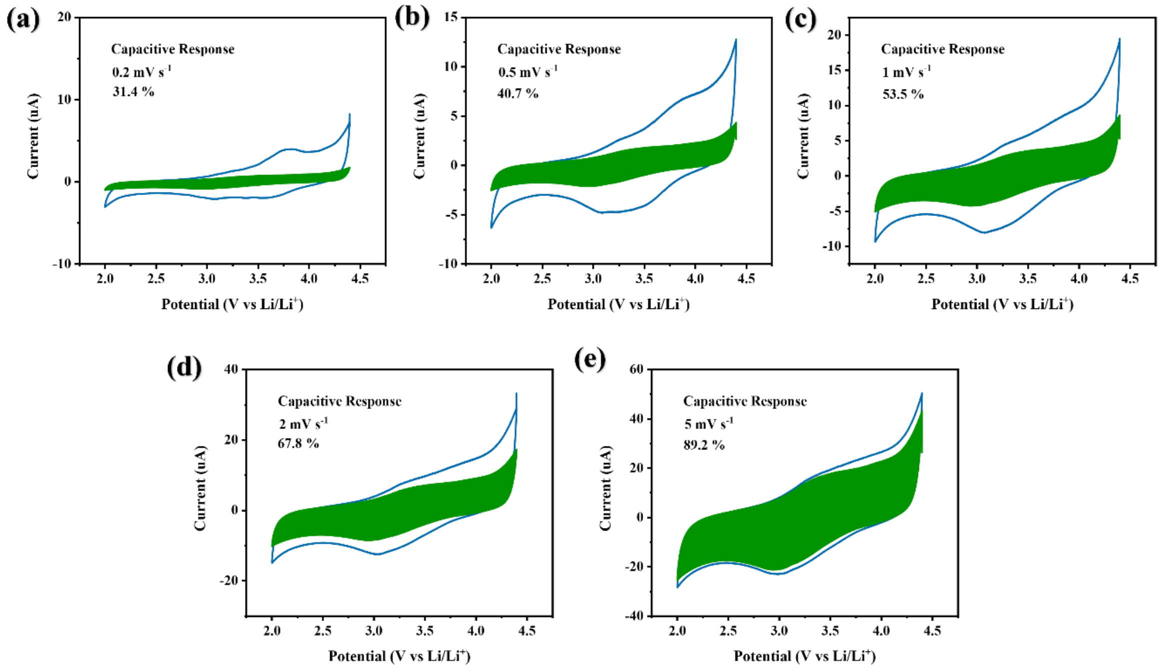

Figure S20. CV curves of ex-PNA cathode and corresponding capacitive contributions at scan rates of (a) 0.2 mV s^{-1}, (b) 0.5 mV s^{-1}, (c) 1.0 mV s^{-1}, (d) 2.0 mV s^{-1}, (e) 5 mV s^{-1}.

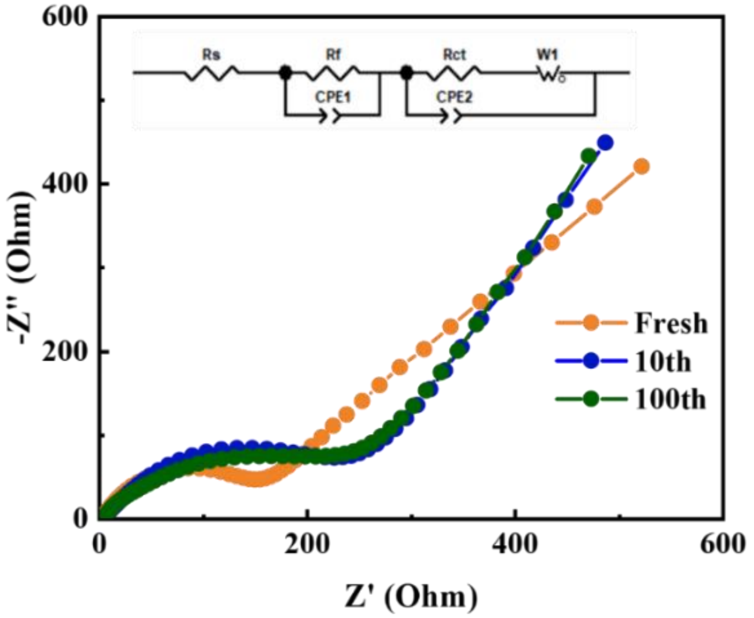

Figure S21. Impedance evolution with cycling of ex-PNA cathode.

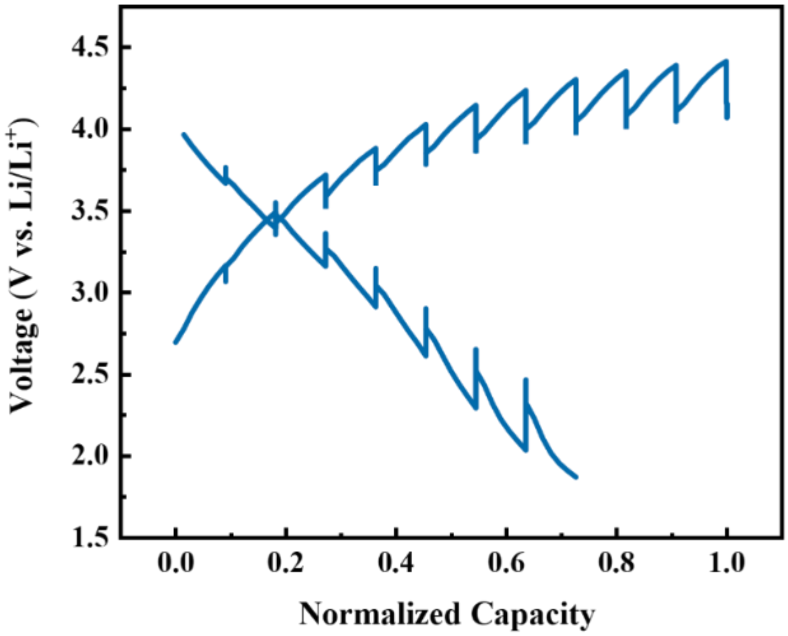

Figure S22. GITT curves of NA/CMK-3 cathode as a function of normalized capacity at a current density of 0.2 A g^{-1}.

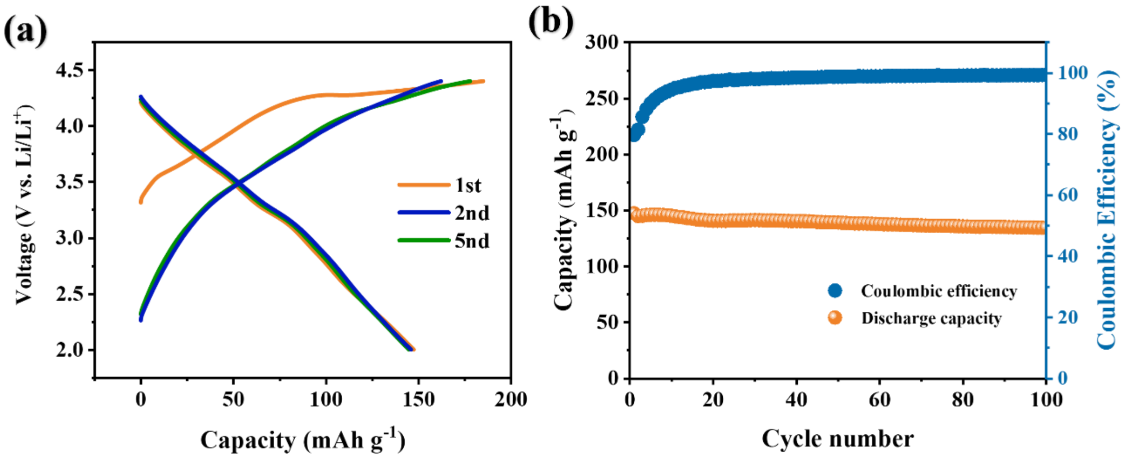

Figure S23. (a) The GCD profiles of NA/CMK-3 cathode, corresponding (b) cycle performance under 0.2 A g^{-1} in the voltage range of 2.0-4.4 V at -15 °C.

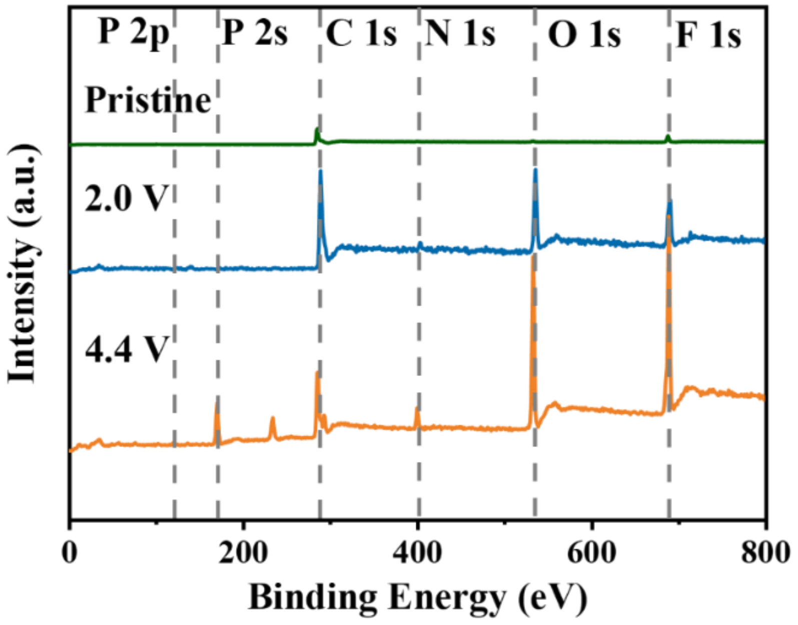

Figure S24. Full survey spectra of the NA/CMK-3 cathode at different charge/discharge state.

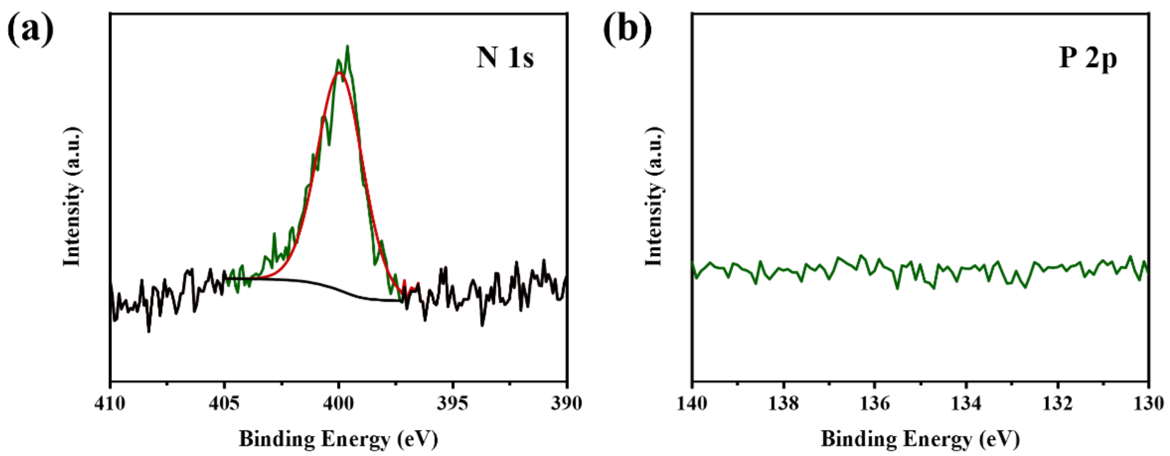

Figure S25. The high-resolution (a) N 1s and (b) P 2p of NA/CMK-3 cathode.

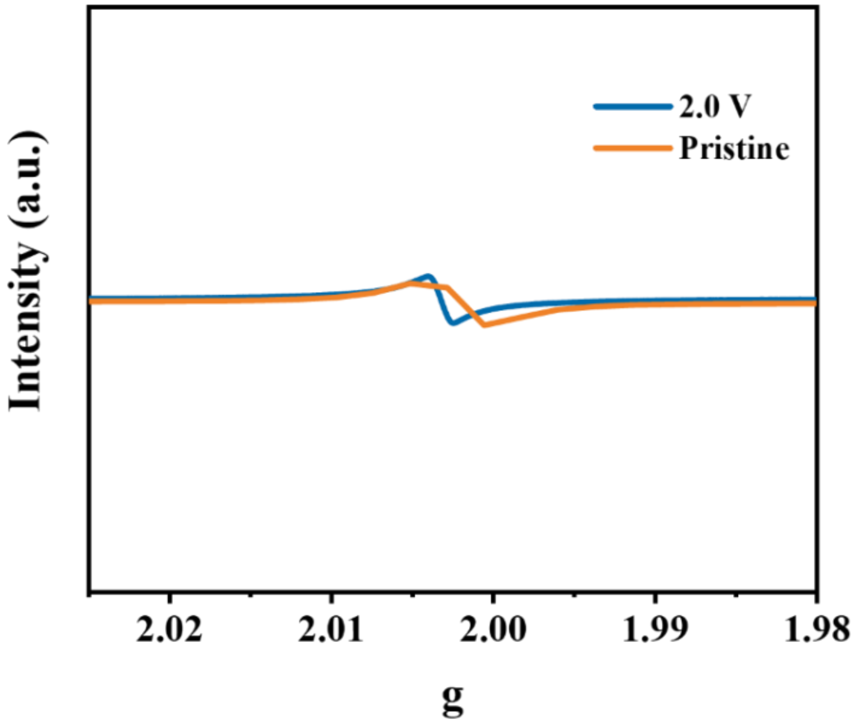

Figure S26. EPR spectra of NA/CMK-3 cathode at 2.0 V and pristine state.

Table S1. Electrochemical performance comparison of high-voltage lithium-organic batteries.

Cathode	Capacity (mAh g-1)	Cycling stability	Discharge Voltage (V)	Ref.
**NA/CMK-3**	**130.1**	**71.3% after 10000 cycles at 5 A g^{-1}**	**3.3**	**Our work**
DCN	37.2	76.2% after 5000 cycles at 2 A g^{-1}	4.0	[1]
Thianthrene	20.3	31.1% after 100 cycles at 0.073 A g^{-1}	4.0	[2]
PVMPT	53.6	93.5% after 10000 cycles at 1.12 A g^{-1}	3.6	[3]
Li2DzAnT	80.0	75.2% after 20 cycles at 0.015 A g^{-1}	3.2	[4]
3PXZ	80.3	80.0% after 500 cycles at 0.65 A g^{-1}	3.7	[5]
YPTPA	88.0	69.7% after 1700 cycles at 2 A g^{-1}	3.6	[6]
PDPPD	84.2	67.7% after 5000 cycles at 20.9 A g^{-1}	3.4	[7]
TCTA	92.3	52.3% after 5000 cycles at 1 A g^{-1}	3.95	[8]
PTMA	111.0	84.8% after 200 cycles at 0.11 A g^{-1}	3.13/3.8	[9]
P-DPPZS	114.1	90.2% after 1000 cycles at 0.74 A g^{-1}	3.1/3.8	[10]
PVK	117.2	93.6% after 50 cycles at 0.02 A g^{-1}	3.9	[11]
TZPZ	125	≈100% after 10000 cycles at 5 A g^{-1}	3.2	[12]
PT-DMPD	128	64.4% after 50 cycles at 0.16 A g^{-1}	3.3	[13]
DMPZ	220	60.0% after 30 cycles at 0.05 A g^{-1}	3.0	[14]
P-DPPZ	130	89.4% after 500 cycles at 0.3 A g^{-1}	3.1/4.3	[15]
ET-PXZ	244	85% after 100 cycles at 0.3 A g^{-1}	3.2/3.8	[16]
PAH	40	92.6% after 960 cycles at 0.2 A g^{-1}	4.0	[17]
P3OT	100	90.7% after 200 cycles at 0.05 A g^{-1}	3.7	[18]
Pth	17.5	71.4% after 100 cycles at 0.16 A g^{-1}	3.6	[19]

Table S2. The fitting EIS data of cathodes after different cycles.

**Cathodes**	cycles	R_s (Ω)	R_f (Ω)	R_{ct} (Ω)
**NA/CMK-3** (Figure 4d)	Fresh	3.14	158.2	164.6
	10th	3.65	149.7	158.2
	100th	4.38	155.3	167.3
**Ex-PNA** (Figure S20)	Fresh	3.94	120.8	170.2
	10th	5.12	130.1	282.1
	100th	4.43	122.4	287.4

Table S3. Low-temperature performance comparison of organic batteries in our work and previous reports.

Cathode/anode	Temperature (°C)	Cycling stability	Ref.
**NA-CMK-3/Li**	**-15**	**81.3% after 45,000 cycles at 5 A g^{-1}**	**Our work**
Me_2BBQ/Li	0	92.3% after 50 cycles at 0.06 A g^{-1}	[20]
PEO-NGPE/Li	-70	90.0% after 500 cycles at 0.4 A g^{-1}	[21]
PI/Li/Li	-70	75.2% after 100 cycles at 0.25 A g^{-1}	[22]
PTPAN/Li	-10	99.8% after 200 cycles at 0.2 A g^{-1}	[23]
PNA-V_2O_5/Zn	-20	71.7% after 2000 cycles at 2 A g^{-1}	[24]
PNA/Zn	-70	≈100% after 2000 cycles at 0.2 A g^{-1}	[25]
Poly(1,5-NAPD)-AC/Zn	0	90% after 3000 cycles at 7 A g^{-1}	[26]
Poly(1,5-DHN)-AC/Zn	0	94% after 3000 cycles at 3 A g^{-1}	[26]
P($4VC_{86}$-stat-SS_{14})/Zn	-35	97.4% after 50 cycles at 0.6 A g^{-1}	[27]
NCA/Li	-20	95.5% after 450 cycles at 0.057 A g^{-1}	[28]
$LiNi_{0.5}Co_{0.2}Mn_{0.3}O_2$/graphite	-10	81.87% after 50 cycles at 0.3 A g^{-1}	[29]
$LiNi_{0.5}Co_{0.2}Mn_{0.3}O_2$/graphite	-20	93.0% after 50 cycles at 0.75 A g^{-1}	[30]
LTO/Li	-40	≈100% after 60 cycles at 0.2 A g^{-1}	[31]

References

[1] Z. Wang, J. Yang, Z. Chen, L. Ye, Y. Xu, *ChemSusChem* **2021**, 14, 4573.

[2] M. E. Speer, M. Kolek, J. J. Jassoy, J. Heine, M. Winter, P. M. Bieker, B. Esser, *Chemical Communication* **2015**, 51, 15261.

[3] M. Kolek, F. Otteny, P. Schmidt, C. Mueck-Lichtenfeld, C. Einholz, J. Becking, E. Schleicher, M. Winter, P. Bieker, B. Esser, *Energy & Environmental Science* **2017**, 10, 2334.

[4] E. Deunf, P. Moreau, E. Quarez, D. Guyomard, F. Dolhem, P. Poizot, *Journal of Materials Chemistry A* **2016**, 4, 6131.

[5] K. Lee, I. E. Serdiuk, G. Kwon, D. J. Min, K. Kang, S. Y. Park, J. E. Kwon, *Energy & Environmental Science* **2020**, 13, 4142.

[6] C. Zhang, X. Yang, W. Ren, Y. Wang, F. Su, J. X. Jiang, *Journal of Power Sources* **2016**, 317, 49.

[7] F. Obrezkov, A. F. Shestakov, V. F. Traven, K. Stevenson, P. Troshin, *Journal of Materials Chemistry A* **2019,** 7, 11430.

[8] C. Zhao, Z. Chen, W. Wang, P. Xiong, B. Li, M. Li, J. Yang, Y. Xu, *Angewandte Chemie International Edition* **2020**, 59, 11992.

[9] J. K. Kim, G. Cheruvally, J. H. Ahn, Y. G. Seo, D. S. Choi, S. H. Lee, C. E. Song, *Journal of Industrial & Engineering Chemistry* **2008**, 14, 371.

[10] G. Dai, Y. Liu, Z. Niu, P. He, Y. Zhao, X. Zhang, H. Zhou, *Matter* **2019**, 1, 945.

[11] M. Yao, H. Senoh, T. Sakai, T. Kiyobayashi, *Journal of Power Sources* **2012**, 202, 364.

[12] W. Ma, L. e. Luo, P. Dong, P. Zheng, Y. Cao, *Advanced Functional Materials* **2021**, 2105027.

[13] B. M. Peterson, D. Ren, L. Shen, Y. C. M. Wu, B. P. Fors, *ACS Applied Energy Materials* **2018**, 1, 3560.

[14] M. Lee, J. Hong, B. Lee, K. Ku, K. Kang, *Green Chemistry* **2017**, 19, 2980.

[15] G. Dai, X. Wang, Y. Qian, Z. Niu, Z. Xi, J. Ye, Z. Yu, X. Zhang, *Energy Storage Materials* **2018**, 16, 236.

[16] S. Lee, K. Lee, K. Ku, J. Hong, S. Y. Park, J. E. Kwon, K. Kang, *Advanced Energy Materials* **2020**, 10, 2001635.

[17] I. A. Rodriguez Perez, Z. Jian, P. K. Waldenmaier, J. W. Palmisano, R. S. Chandrabose, X. Wang, M. M. Lerner, R. G. Carter, X. Ji, *Acs Energy Letters* **2017**, 1, 719.

[18] T. Li, L. Wang, J. Li, *Chemical Engineering Journal* **2022**, 442, 136232.

[19] K. S. R. A, Y. L. B, K. S. H. C, M. G. K. D, *Materials Chemistry and Physics* **2004**, 84, 380.

[20] S. Bai, B. Kim, C. Kim, O. Tamwattana, H. Park, J. Kim, D. Lee, K. Kang, *Nature Nanotechnology* **2021**, 16, 77.

[21] M. Li, J. Yang, Y. Shi, Z. Chen, P. Bai, H. Su, P. Xiong, M. Cheng, J. Zhao, Y. Xu, *Advanced Materials* **2022**, 34, 2107226.

[22] X. Dong, Y. Lin, P. Li, Y. Ma, J. Huang, D. Bin, Y. Wang, Y. Qi, Y. Xia, *Angewandte Chemie International Edition* **2019**, 58, 5623.

[23] J. Qin, Q. Lan, N. Liu, F. Men, X. Wang, Z. Song, H. Zhan, *iScience* **2019**, 15, 16.

[24] N. Chang, T. Li, R. Li, S. Wang, Y. Yin, H. Zhang, X. Li, *Energy & Environmental Science* **2020**, 13, 3527.

[25] Q. Zhang, Y. Ma, Y. Lu, L. Li, F. Wan, K. Zhang, J. Chen, *Nature Communication* **2020**, 11, 4463.
[26] N. Wang, R. Zhou, H. Li, Z. Zheng, W. Song, T. Xin, M. Hu, J. Liu, *ACS Energy Letters* **2021**, 6, 1141.
[27] N. Patil, C. Cruz, D. Ciurduc, A. Mavrandonakis, J. Palma, R. Marcilla, *Advanced Energy Materials* **2021**, 11, 2100939.
[28] X. Fan, X. Ji, L. Chen, J. Chen, T. Deng, F. Han, J. Yue, N. Piao, R. Wang, X. Zhou, X. Xiao, L. Chen, C. Wang, *Nature Energy* **2019**, 4, 882.
[29] R. Guo, Y. Che, G. Lan, J. Lan, J. Li, L. Xing, K. Xu, W. Fan, L. Yu, W. Li, *ACS Appl Mater Interfaces* **2019**, 11, 38285.
[30] B. Liao, H. Li, M. Xu, L. Xing, Y. Liao, X. Ren, W. Fan, L. Yu, K. Xu, W. Li, *Advanced Energy Materials* **2018**, 8, 1800802.
[31] J. Xu, X. Wang, N. Yuan, J. Ding, S. Qin, J. M. Razal, X. Wang, S. Ge, Y. Gogotsi, *Energy Storage Materials* **2019**, 23, 383.
